# Multifunctional Silk Fibroin/Carbon Nanofiber Scaffolds for In Vitro Cardiomyogenic Differentiation of Induced Pluripotent Stem Cells and Energy Harvesting from Simulated Cardiac Motion

**DOI:** 10.1021/acsami.3c08601

**Published:** 2023-08-29

**Authors:** Yiğithan Tufan, Hayriye Öztatlı, Doga Doganay, Arda Buyuksungur, Melih Ogeday Cicek, İpek Tuğçe Döş, Çağla Berberoğlu, Husnu Emrah Unalan, Bora Garipcan, Batur Ercan

**Affiliations:** † Department of Metallurgical and Materials Engineering, Middle East Technical University, Çankaya, 06800 Ankara, Turkey; ‡ Institute of Biomedical Engineering, Boğaziçi University, 34684 İstanbul, Turkey; § Department of Basic Medical Sciences, Faculty of Dentistry, Ankara University, 06560 Ankara, Turkey; ∥ Biomedical Engineering Program, Middle East Technical University, Çankaya, 06800 Ankara, Turkey; ⊥ BIOMATEN, Center of Excellence in Biomaterials and Tissue Engineering, Middle East Technical University, Çankaya, 06800 Ankara, Turkey

**Keywords:** silk fibroin, enzymatic degradation, cardiac patch, stem cell, triboelectric nanogenerators

## Abstract

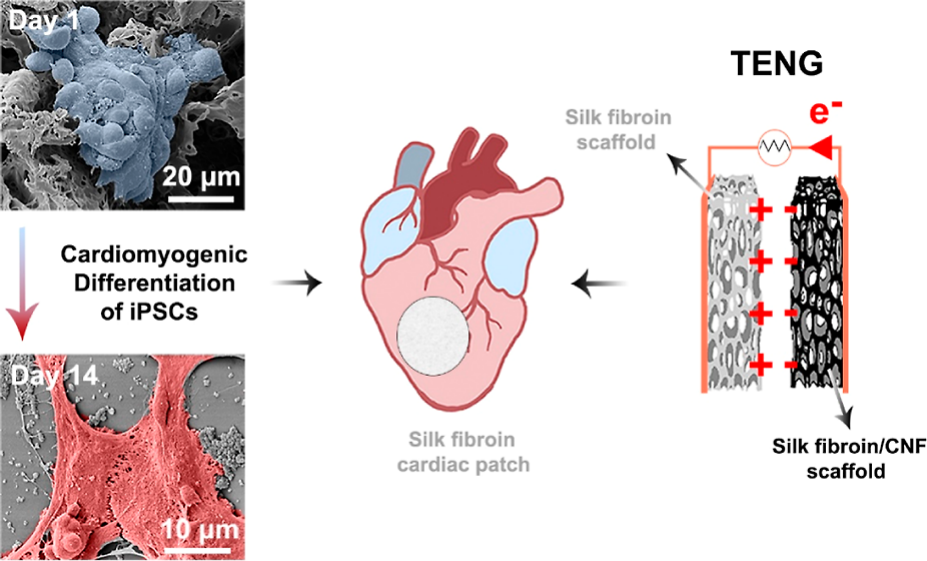

In this proof-of-concept study, cardiomyogenic differentiation of induced pluripotent stem cells (iPSCs) is combined with energy harvesting from simulated cardiac motion in vitro. To achieve this, silk fibroin (SF)-based porous scaffolds are designed to mimic the mechanical and physical properties of cardiac tissue and used as triboelectric nanogenerator (TENG) electrodes. The load-carrying mechanism, β-sheet content, degradation characteristics, and iPSC interactions of the scaffolds are observed to be interrelated and regulated by their pore architecture. The SF scaffolds with a pore size of 379 ± 34 μm, a porosity of 79 ± 1%, and a pore interconnectivity of 67 ± 1% upregulated the expression of cardiac-specific gene markers TNNT2 and NKX2.5 from iPSCs. Incorporating carbon nanofibers (CNFs) enhances the elastic modulus of the scaffolds to 45 ± 3 kPa and results in an electrical conductivity of 0.021 ± 0.006 S/cm. The SF and SF/CNF scaffolds are used as conjugate TENG electrodes and generate a maximum power output of 0.37 × 10^–3^ mW/m^2^, with an open-circuit voltage and a short circuit current of 0.46 V and 4.5 nA, respectively, under simulated cardiac motion. A novel approach is demonstrated for fabricating scaffold-based cardiac patches that can serve as tissue scaffolds and simultaneously allow energy harvesting.

## 1 Introduction

Current treatments for heart failure often rely on heart transplantation or the implantation of mechanical devices, both of which possess notable disadvantages.^1^ Heart transplants have limited availability, carry the risk of rejection, and force patients to use long-term immunosuppressants.^2^ Battery-operated mechanical devices, such as ventricular assist devices and cardioverter defibrillators, suffer from limited battery life, formation of blood clots, and monitoring and maintenance requirements.^3,4^ As an alternative approach, regenerative therapies offer the use of induced pluripotent stem cells (iPSCs) to repair damaged cardiac tissue.^5^ This approach can reduce the risk of immune rejection and eliminate the need for immunosuppressive drugs. However, using iPSCs in cardiac regeneration is still being researched to provide an optimal environment for their growth and cardiomyogenic differentiation.^6^ To address this challenge, studies focused on the development of scaffold-based cardiac patches, which provide a porous structure for iPSCs to adhere, grow, and differentiate.^7^

Silk fibroin (SF) is a biocompatible protein-based material that can be processed to fabricate porous scaffolds.^8^ The pore architecture, particularly the pore size, porosity, and pore interconnectivity of the SF scaffolds, could be altered to achieve optimum properties for cardiac tissue regeneration.^9^ It is known that the pore architecture determines the mechanical, physical, biological, and degradation characteristics of the scaffolds.^10^ Thus, precise control over the pore architecture will allow the fabrication of a scaffold that can mimic cardiac tissue. Still, more research is needed to explore the simultaneous effects of pore size, porosity, and pore interconnectivity on scaffold properties for cardiac tissue regeneration. That said, the lack of electrical conductivity is a critical limitation of SF-based scaffolds used as cardiac patches.^11^ Incorporating electrically conductive, high-aspect-ratio carbon nanofibers (CNFs) can address the aforementioned shortcoming. Besides, CNFs provide further opportunities to adjust the mechanical properties and degradation characteristics of the SF scaffolds.^12^

Aside from their potential for cardiac regeneration, SF/CNF scaffolds also offer opportunities for energy harvesting. Triboelectric nanogenerators (TENGs) are a promising technology for converting mechanical energy into electrical energy.^13^ For cardiac applications, the heart’s pulsatile nature offers a potential source of mechanical energy that can be converted into electrical energy when cardiac patches are used as TENGs.^4^ In the literature, all TENG systems designed to harvest energy from cardiac motion were closed systems, mostly encapsulated in bioinert cases, and none of them designed TENG electrodes that simultaneously supported iPSC growth and cardiomyogenic differentiation.^14−20^

**Figure 1:**
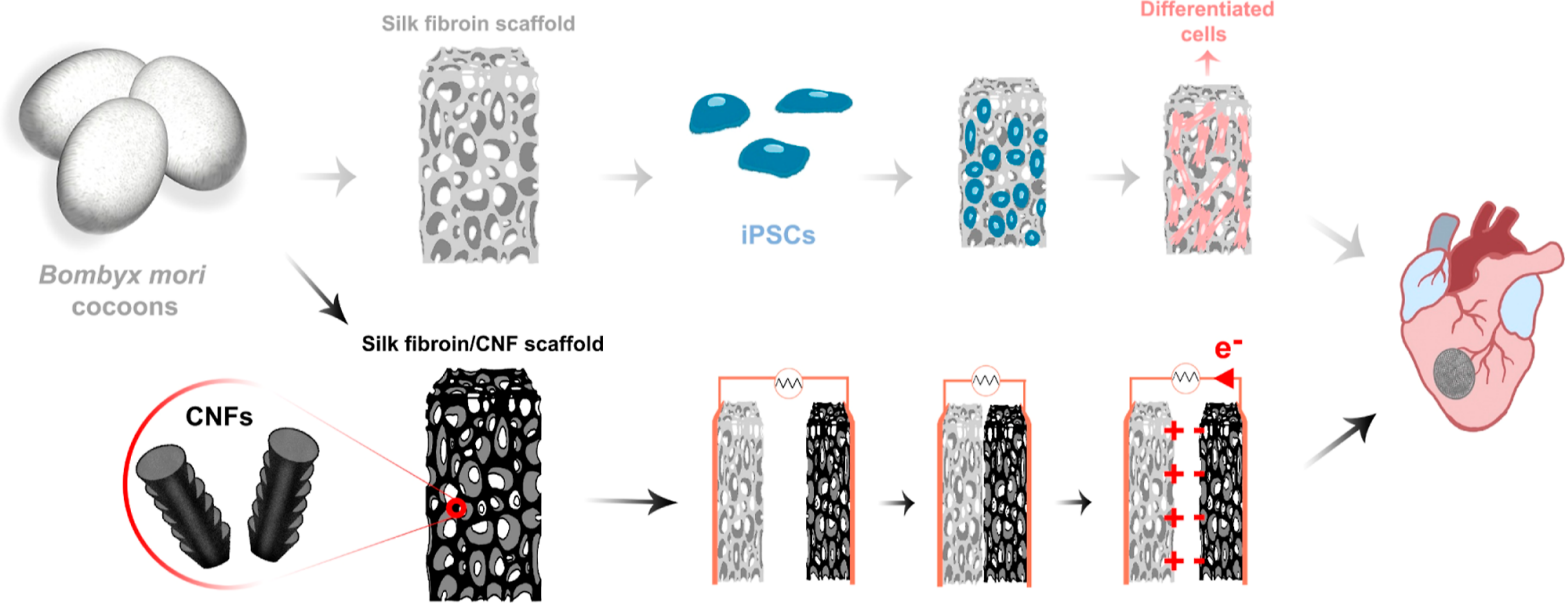
Schematic of the potential use of SF and SF/CNF scaffolds for cardiomyogenic differentiation of iPSCs and energy harvesting from simulated cardiac motion in vitro.

This article aims to comprehensively analyze the simultaneous effect of pore size, porosity, pore interconnectivity, and CNF content on the mechanical, biological, enzymatic degradation, and electrical properties of SF/CNF scaffolds for cardiac patch applications. Toward this goal, we fabricated porous SF and SF/CNF scaffolds (having 0 and 10 wt % CNFs) using two different porogen sizes (50–90 and 350–425). The scaffold that best mimics the native cardiac tissue and induces cardiomyogenic differentiation of iPSCs was evaluated for its energy-harvesting potential. Our approach is summarized in Figure 1, where the pore architecture of SF and SF/CNF scaffolds is designed for in vitro cardiomyogenic differentiation of iPSCs and energy harvesting from a TENG device. Although still in its infancy, the presented proof-of-concept study will contribute to the current literature for the development of multifunctional scaffolds that integrate regenerative therapy and harvest energy.

## 2 Results and Discussion

### 2.1 Characterization of the Scaffolds

The dimensions (thickness and diameter) of SF and SF/CNF scaffolds were optimized for use as cardiac patches. Photographs of SF350-0 and SF350-10 having a 6 cm diameter and ∼1 mm thickness are shown in Figure S1. The figure also shows that the scaffolds are flexible and durable enough for easy handling and suturing.

**Figure 2:**
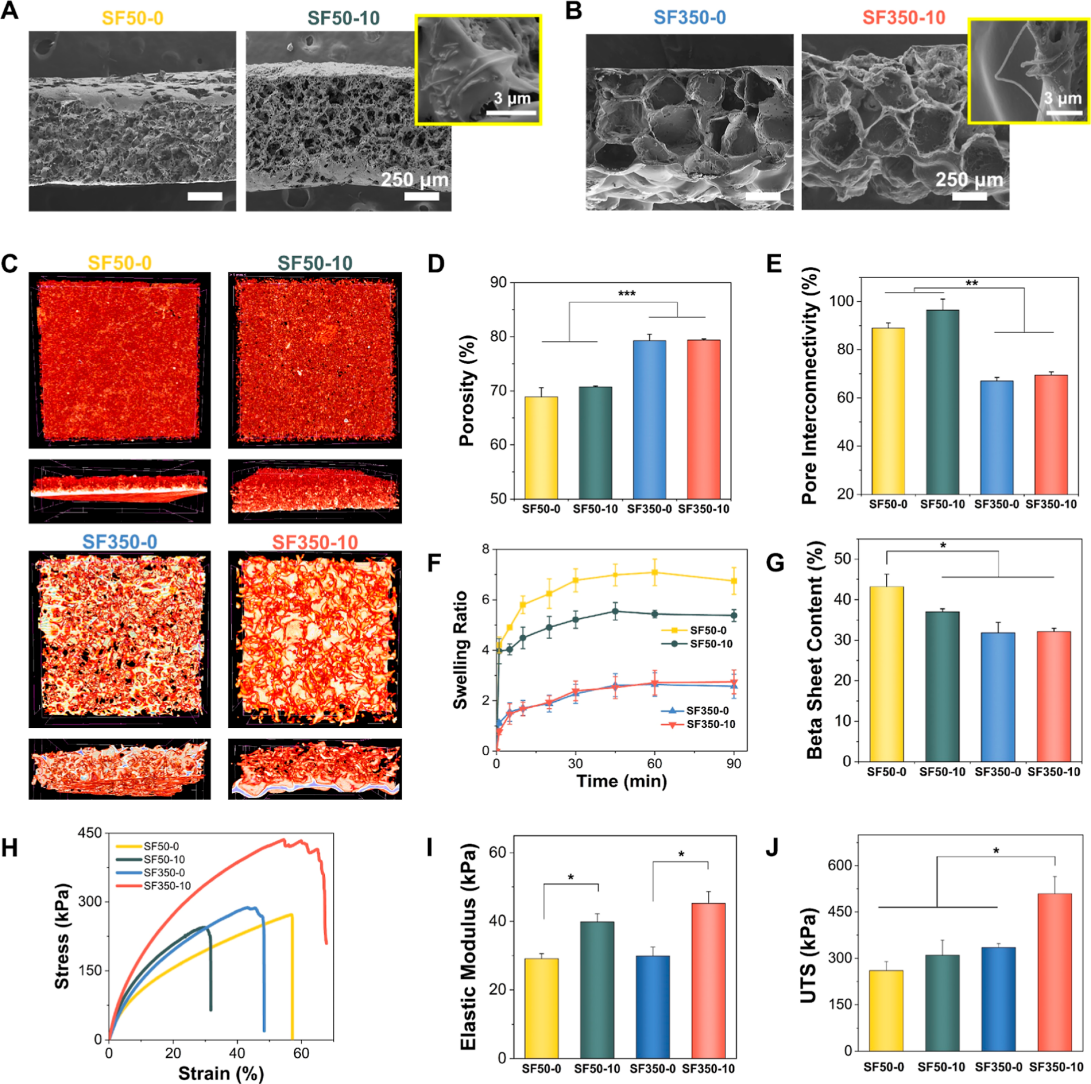
Characterization of SF and SF/CNF scaffolds. SEM images of (A) SF50-0 and SF50-10 and (B) SF350-0 and SF350-10. (C) Micro-CT images, (D) porosity, (E) pore interconnectivity values, (F) swelling ratios, (G) β-sheet percentages, (H) stress–strain curves, (I) elastic moduli, and (J) UTS values of SF50-0, SF50-10, SF350-0, and SF350-10. *p < 0.05, **p < 0.01, and ***p < 0.001.

The pore morphologies of the scaffolds were characterized by scanning electron microscopy (SEM). Cross-sectional SEM images of SF50 scaffolds and SF350 scaffolds are provided in Figure 2A,B, respectively. The scaffolds were fabricated by the salt leaching method, where the average pore size is determined by the size of sodium chloride (NaCl) particles. The average pore size of SF50-0, SF50-10, SF350-0, and SF350-10 was measured as 74 ± 19, 78 ± 11, 379 ± 34, and 392 ± 34 μm, respectively. The presence of CNFs had no apparent effect on the pore morphology of the scaffolds. CNFs were apparent on SF50-10 and SF350-10 surfaces without showing any evidence of agglomeration (see the insets in Figure 2A,B). Micro-CT images (Figure 2C) confirmed the porous architecture of the scaffolds, where the porosity and pore interconnectivity of the scaffolds were quantified. Porosity of SF50-0, SF50-10, SF350-0, and SF350-10 was calculated as 69 ± 2, 71 ± 0.2, 79 ± 1, and 79 ± 0.2%, respectively (Figure 2D). These results showed that the use of NaCl particles with a size range of 350–425 μm leads to higher porosity than the use of 50–90 μm NaCl particles. The percent pore interconnectivity of the scaffolds is provided in Figure 2E. The use of 50–90 μm NaCl particles provided a higher percentage of interconnected pores in SF50-0 (88 ± 2%) and SF50-10 (96 ± 5%) compared to the SF350-0 (67 ± 1%) and SF350-10 (69 ± 1%) scaffolds. This difference in the pore interconnectivity of the scaffolds can be attributed to differences in the packing efficiency of smaller and larger NaCl particles.

The decreased ability of larger NaCl particles to pack more efficiently can result in more air being trapped between the particles. This, in turn, might result in increased porosity and decreased pore interconnectivity for the SF350 scaffolds. These results were in accord with the swelling ratios of the scaffolds, as provided in Figure 2F. The swelling ratios of SF50-0 and SF50-10 after 60 min were measured to be around 7 and 5, respectively. On the other hand, SF350-0 and SF350-10 both had a similar swelling ratio of around 2.5 after 60 min. The high pore interconnectivity led to increased diffusion of water molecules within the SF50 scaffolds, which increased swelling ratios compared to the SF350 scaffolds.

Secondary structural components of the scaffolds were characterized using deconvoluted FTIR spectra at amide I regions (see Figure S2). The β-sheet content of the scaffolds is compared in Figure 2G. SF50-0 contained a higher amount of β-sheets than the other scaffolds (*p* < 0.05), whereas no statistically significant difference was observed between the β-sheet content of SF50-10, SF350-0, and SF350-10 (*p* > 0.05). The crystallization results could be correlated with the swelling ratios of the scaffolds. The higher pore interconnectivity of SF50-0 could lead to more efficient penetration of methanol into the 3D structure of scaffolds than SF350, resulting in more β-sheet formation in SF50-0. Though there was no statistically significant difference between the pore interconnectivity of SF50-0 and SF50-10, the former had a higher β-sheet content than the latter. This finding was in line with our previous results.^12^ The incorporation of CNFs decreased the efficacy of β-sheet formation in SF50-10. One of the reasons for the decreased crystallinity of SF50-10 could be the inhibition of the SF-methanol interactions due to secondary bonding between the CNFs’ functional groups (mainly −OH and −COOH) and SF. Another potential reason could be the increased fraction of aggregated strands in SF50-10 than in SF50-0 (*p* < 0.05) (see Figure S2). The surfaces of CNFs can provide favorable sites for the aggregation of SF polypeptide chains and potentially inhibit the formation of β-sheets.

The results showed that while CNFs reduced the β-sheet content and swelling ratio of SF50-10, this effect was not observed in SF350-10. This finding could be attributed to the NaCl-methanol interactions during the crystallization of SF scaffolds. The smaller-sized NaCl particles had a higher surface area-to-volume ratio than the larger ones. The former allowed a higher amount of NaCl to dissolve in methanol and more surface area for crystallization. It can be speculated that smaller NaCl particles dissolve faster and at a higher extent in methanol compared to larger NaCl particles. As mentioned previously, smaller-sized NaCl particles also provided higher interconnectivity for SF50 scaffolds, leading to easier diffusion of methanol across the SF50 scaffolds. Because of these reasons (the faster/higher dissolution of NaCl particles and higher interconnectivity), all CNFs throughout the SF50-10 contributed to the prevention of crystallization. On the other hand, larger-sized NaCl particles dissolved less and more slowly than smaller-sized NaCl particles. It might leave undissolved NaCl particles. These particles, as well as the less interconnected pores of SF350 scaffolds, might act as physical barriers and slow the diffusion of methanol across the scaffolds. As a result, CNFs did not significantly affect crystallization in SF350 scaffolds, as the number of CNFs that prevent β-sheet formation was limited.

Stress–strain curves, elastic moduli, and ultimate tensile strength (UTS) values of the scaffolds are given in Figure 2H–J, respectively. The elastic moduli of SF50-0 (29 ± 1 kPa) and SF350-0 (30 ± 3 kPa) increased to 40 ± 2 and 45 ± 3 kPa, respectively, upon the incorporation of 10 wt % CNFs. The UTS values of the scaffolds were measured to be 260 ± 29, 310 ± 49, 335 ± 13, and 509 ± 55 kPa for SF50-0, SF50-10, SF350-0, and SF350-10, respectively (Figure 2J). A statistically significant increase in elastic modulus values was obtained with the inclusion of CNFs, independent of the pore size of the scaffold (*p* < 0.05). That said, there was no increase in the UTS of SF50-10 compared to SF50-0. However, the UTS of SF350-10 increased significantly, compared to SF350-0, upon the incorporation of CNFs (Figure 2J). It is also worth noting that SF50-0 and SF350-0 have comparable elastic moduli and UTS values. Similarly, the mechanical properties of SF50-10 and SF350-10 did not differ significantly (*p* > 0.05).

In principle, increased porosity decreases the mechanical properties of the scaffolds. However, SF350-0 had around 10% higher porosity than SF50-0, yet these two scaffolds had similar mechanical properties. Our results suggested that pore interconnectivity and β-sheet content of SF scaffolds were also critical factors influencing their mechanical properties. Indeed, high interconnectivity is detrimental to load-carrying capacity but also leads to increased β-sheet content. Another critical factor that affects the load-bearing capacity of the scaffolds is the thickness of the pore walls, which depends on the scaffolds’ porosity and pore size. Therefore, the overall effect of the aforementioned factors should be considered together to understand the final mechanical properties of the scaffolds. For simplicity, a schematic drawing based on the pore size, porosity, and pore interconnectivity of the scaffolds was prepared (see Figure S3). The arbitrarily selected, equal load-carrying volumes were highlighted on the schematic for both scaffolds (Figure S3B,D). SF50-0 had a significant porous fraction within this volume, with a smaller pore size and higher pore interconnectivity than SF350-0. This pore architecture led to increased β-sheet content in SF50-0. As the formation of β-sheets enhanced the mechanical properties, it compensated for the detrimental effect of the pore interconnectivity on the load-carrying capacity of SF50-0. On the other hand, in SF350-0, the smallest load-carrying volume (due to having thick pore walls, as shown in Figure S3D) was composed of bulk SF without any porosity. This pore architecture was advantageous in load-carrying but resulted in less β-sheet content. Taken together, having less porosity and higher β-sheet content favored SF50-0 while having less interconnected pores favored SF350-0 in terms of mechanical properties. As a result, SF50-0 and SF350-0 had comparable elastic moduli and UTS values.

For the scaffolds having CNFs (SF50-10 and SF350-10), the high elastic modulus of CNFs was observed to increase the elastic modulus of the scaffolds, independent of their pore sizes, compared to their CNF-free counterparts. In terms of UTS, CNFs were advantageous only for SF350 scaffolds. That is, incorporating CNFs increased the UTS of SF350-10 compared to SF350-0, yet they did not cause a significant increase for the SF50 scaffolds (*p* > 0.05). Considering that the β-sheet content of SF50-10 and SF350-10 were similar, changes in the UTS values could be attributed to the scaffolds’ load-carrying mechanisms. The discussion above, which relates the porous microstructure of SF50-0 and SF350-0 to their mechanical properties, could apply to SF50-10 and SF350-10. For SF350-10, the applied load was carried by relatively thick pore walls. In SF50-10, the load was carried by thin pore walls surrounded by highly interconnected smaller pores. The results indicated that the effect of CNFs on the elastic modulus and UTS of the scaffolds depended on their porous architecture. To explain the changes in the elastic modulus of the scaffolds, schematics showing SF50-10 (Figure S4A) and SF350-10 (Figure S4B) in the elastic region were drawn. In the elastic region, almost all CNFs participated in carrying the load for both scaffolds and improved their elastic modulus. However, beyond the elastic region, the exact amount of strain led to earlier failure in the thinner pore walls of SF50-10 compared to SF350-10. Once pore walls fail, the CNFs in the failed regions no longer carry the applied load and are unable to reinforce the SF matrix. Since the thinner pore walls in SF50-10 failed at earlier strain levels, CNFs incorporated into SF50-10 did not enhance its UTS value.

In addition to the mechanical properties, it is crucial to design a scaffold that mimics the electrical conductivity of the cardiac tissue. Scaffolds should enable the transmission of electrical signals between the cells and the scaffold, which is important for proper cardiac tissue functioning. Frequency-dependent impedance behavior of the SF film, SF50-0, and SF350-0 scaffolds demonstrated that they act as insulators with very small differences in the ionic conductivity, as shown in Figure S5A. The porosity of the samples was calculated according to their dielectric constants.^21^ For these calculations, SF and air were considered as matrix and filler, respectively. The results are shown in Figure S5B. The porosity values of SF50-0 and SF350-0 were calculated as 70 ± 3 and 79 ± 2%, respectively. Notably, the results perfectly matched the porosity values obtained from the micro-CT analysis (Figure 2D).

Incorporating CNFs significantly altered the electronic behavior of the samples, as shown in Figure S5. The electrical conductivities of the SF/CNF film, SF50-10, and SF-350-10 scaffolds were measured as 0.728 ± 0.031, 0.023 ± 0.003, and 0.021 ± 0.006 S/cm, respectively (Figure S5D). These values are similar to those of cardiac muscle (between 0.0006 and 0.004 S/cm^22^). It should be noted that the pore architecture did not affect the electrical conductivity of the scaffolds, where SF50-10 and SF350-10 had similar electrical conductivity values.

### 2.2 Enzymatic Degradation of the Scaffolds

**Figure 3:**
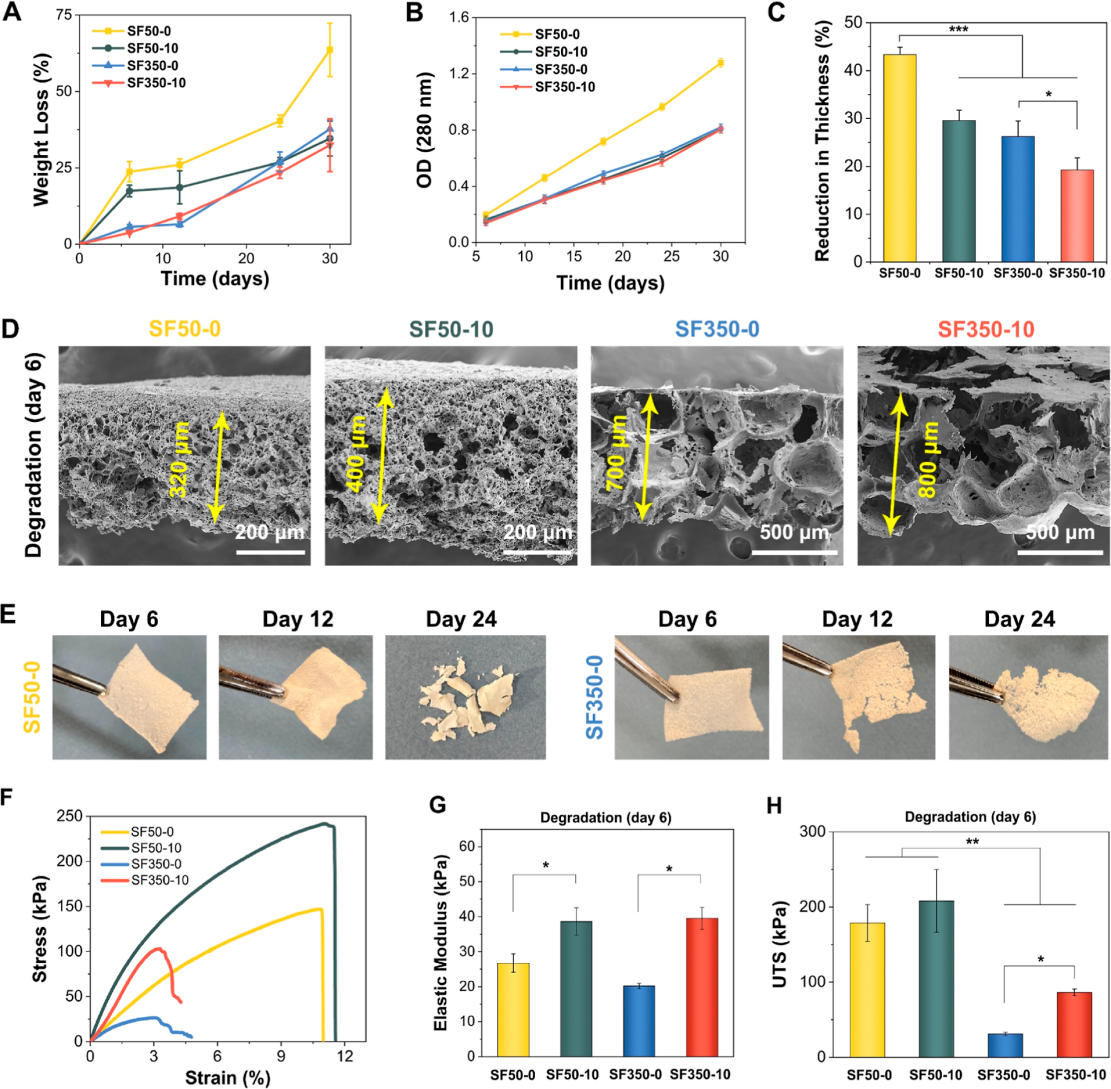
Enzymatic degradation of SF and SF/CNF scaffolds. (A) Weight loss percentages of SF50-0, SF50-10, SF350-0, and SF350-10 up to 30 days of incubation. (B) Optical density values of the enzyme solutions obtained at the designated time points. (C) Reduction in thickness percentages and (D) SEM images of the scaffolds after 6 days of incubation. (E) Visual investigation of SF50-0 and SF350-0 at the 6th, 12th, and 24th days of incubation. (F) Stress–strain curves, (G) elastic moduli, and (H) UTS values of the scaffolds on the 6th day of degradation. *p < 0.05, **p < 0.01, and ***p < 0.001.

The scaffolds were incubated in protease XIV enzyme solutions for up to 30 days to assess their accelerated biodegradation characteristics.^23−25^ Weight loss results are shown in Figure 3A. Among all the scaffolds, the most significant weight loss was observed for SF50-0. A higher degradation rate was observed for SF50-10 than for SF350-0 and SF350-10 at earlier time points until day 12. However, these differences in percent weight loss disappeared after 24 days of incubation. The absorbance values of the enzyme solutions (280 nm) were also compared to assess the concentration differences of the degradation products (Figure 3B). The absorbance values confirmed that the degradation rate of SF50-0 was the highest among all scaffolds. The average reduction-in-thickness percentages of the scaffolds were calculated on the 6th day of incubation (Figure 3C). The thickness of SF50-0 decreased by almost 43%, whereas SF50-10 had a thickness reduction of about 29%. The reduction-in-thickness values of SF350-0 and SF350-10 were about 27 and 19%, respectively. The representative cross-sectional SEM images of the scaffolds on the 6th day of degradation are shown in Figure 3D. See also Figure 3E for photographs of SF50-0 and SF350-0 scaffolds after up to 24 days of enzymatic degradation.

Detailed micro- and macroinvestigations suggested altered degradation patterns for SF50 and SF350 scaffolds, which stem from the differences in their porous architecture. SF50 scaffolds had highly interconnected, relatively smaller pores, which allowed diffusion of the enzyme solution across the entire scaffold and provided a relatively higher surface area for the enzyme-SF interactions. Thus, degradation took place uniformly throughout their volumes. On the other hand, the pore architecture of SF350 scaffolds led to a different degradation pattern, as evident in Figures 3E and S6, respectively. SF350 scaffolds had fewer interconnected pores, surrounded by thick pore walls. It limited their degradation rate by making the diffusion of the enzyme solution more difficult across the scaffolds. In fact, large cracks were apparent in the degraded SF350-0 and SF350-10. This indicated that the degradation proceeded along the pore walls and formed cracks. Once a particular pore wall degraded, it allowed the enzyme solution to access the newly opened pore and promoted further degradation.

The effect of CNFs on the morphological changes of the scaffolds after degradation was also investigated (Figure S6). It was apparent that SF50-10 degraded uniformly throughout its volume after 6, 12, and 24 days of incubation (Figure S6A). It still maintained its structural integrity on day 24. This visual observation was consistent with the quantitative measurements; CNFs decreased the degradation rate of SF50-10 compared to SF50-0. CNFs might prolong the degradation of SF50-10 in two possible ways. First, degraded SF chain residues might remain within the scaffold due to secondary bonding between SF chains and the CNF surfaces. Second, CNFs might act as physical linkages to prevent the separation of degraded SF debris from the scaffold. It should be noted that the CNFs did not cause the same effect in SF350-10. It can be hypothesized that the effect of CNFs on the degradation rate becomes more pronounced when the highly interconnected pores surrounded by thin pore walls allow easy diffusion of the enzyme solution, as in SF50 scaffolds.

Uniaxial tensile tests were performed to assess the effect of degradation on the scaffolds’ mechanical properties after the 6th day of degradation (Figure 3G). Figure 3F shows that the elastic moduli of SF50-0 and SF50-10 after degradation were 27 ± 3 and 39 ± 4 kPa, respectively. These values are similar to the elastic modulus values of their non-degraded counterparts, see Figure 2I. Although the degradation did not cause a significant decrease in the elastic modulus of SF50 scaffolds, it did cause the UTS values to decrease from 260 ± 29 to 179 ± 24 kPa for SF50-0 and from 310 ± 49 to 208 ± 42 kPa for SF50-10 (Figure 3H). The results were in line with the proposed volumetric degradation mechanism of SF50 scaffolds. The degradation caused a uniform material loss throughout their volume. It was therefore reasonable not to expect any changes in their elastic modulus, which is an inherent material property. However, their load-bearing capacity (UTS) decreased after the degradation as the scaffolds’ SF content decreased.

For SF350 scaffolds, the elastic modulus values decreased from 30 ± 3 to 20 ± 1 kPa for SF350-0 and from 45 ± 3 to 40 ± 3 kPa for SF350-10 at the 6th day of degradation. Similarly, their UTS values decreased from 335 ± 13 to 31 ± 2 kPa for SF50-0 and from 509 ± 55 to 86 ± 4 kPa for SF350-10. Unlike the SF50 scaffolds, degradation caused a significant decrease in both the elastic modulus and UTS of the SF350-0 scaffolds. This result could be attributed to their distinct degradation mechanisms. The degradation of SF350 scaffolds caused large cracks in their structure, as discussed above (see Figures 3E and S6B). These cracks could act as stress concentrators and be susceptible to growth even under relatively low tensile loads. That said, after 6 days of degradation, SF350-10 had a significantly higher elastic modulus and UTS than SF350-0. Also, the SEM images of SF350-10 after 12 days of degradation showed that CNFs bridge the degraded SF matrix, see Figure S6B. This indicated that CNFs provided further mechanical support to the degraded SF matrix and helped maintain its physical integrity. We also quantified the amount of secondary structural components in the scaffolds on the 6th day of degradation, and the results are provided in Figure S7. There was no significant difference between the β-sheet content of the scaffolds (*p* > 0.05). Thus, the discussion on the scaffolds’ mechanical properties after degradation was mainly built on their porous architecture and the CNF content.

It is worth noting that the enzymatic degradation of SF scaffolds provides an accelerated model for assessing their degradation characteristics. This method allowed us to understand how scaffold characteristics, such as porous architecture and CNF content, affect their degradation patterns. However, this approach did not precisely mirror the actual in vivo degradation timeline of the scaffolds. In fact, HFIP-derived SF scaffolds were reported to exhibit an in vivo degradation period of over one year.^26^

### 2.3 Viability, Morphology, and Differentiation of iPSCs on SF and SF/CNF Scaffolds

**Figure 4:**
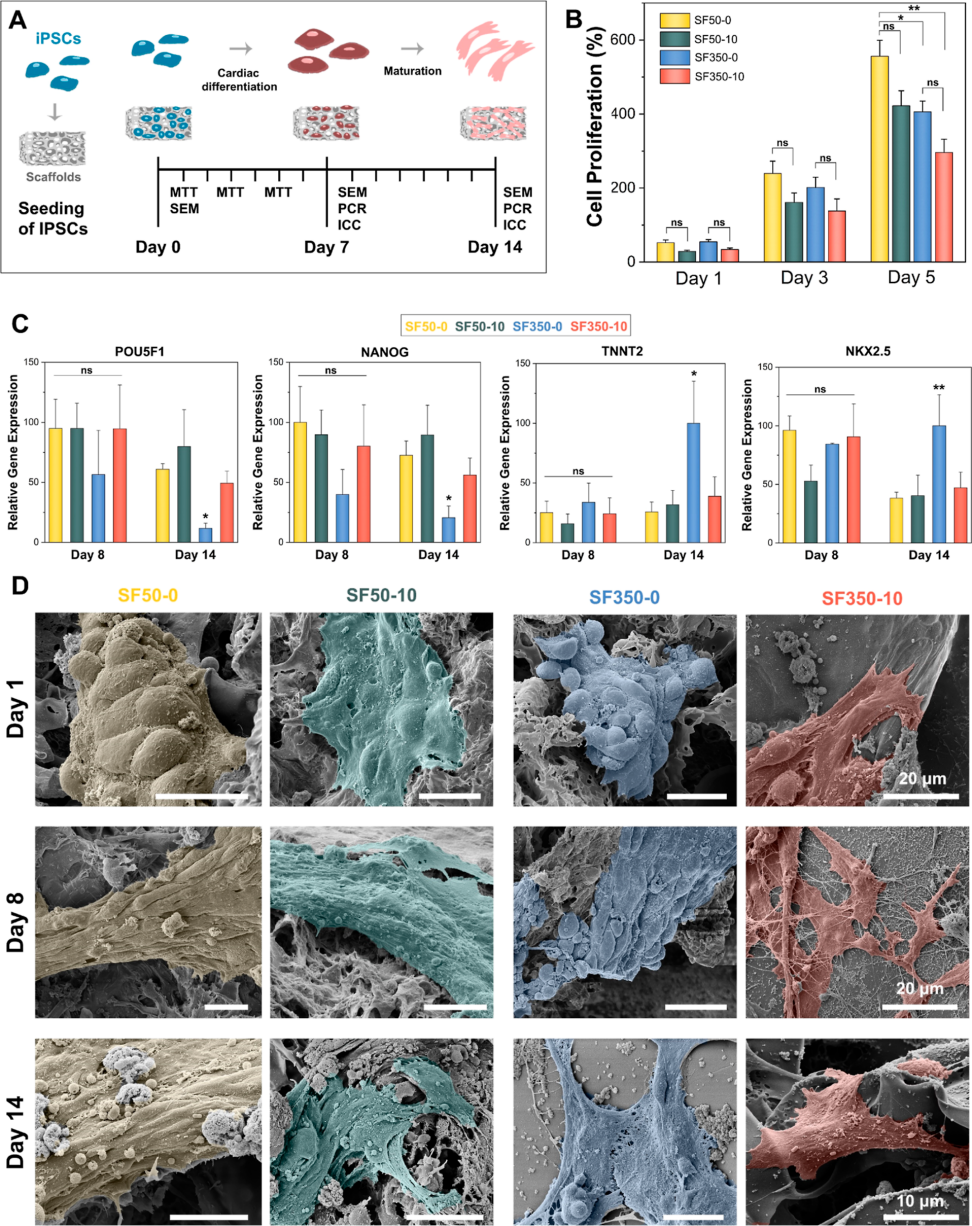
In vitro cardiomyogenic differentiation of iPSCs. (A) Schematic showing the timeline for in vitro iPSC differentiation and biological characterizations. (B) MTT assay results of iPSCs cultured on SF50-0, SF50-10, SF350-0, and SF350-10 up to 5 days of culture. (C) Relative expression levels of pluripotent-specific (Pouf5f1 and Nanog) and cardiac-specific (NKX2.5 and TNNT2) gene markers at the 8th and 14th days of culture. (D) False-colored SEM images of the iPSCs captured on the 1st, 8th, and 14th days of culture on the scaffolds. ns: not significant, *p < 0.05, and **p < 0.01.

The detailed timeline for the in vitro cardiomyogenic differentiation of iPSCs and the conducted biological experiments is sketched in Figure 4A. The viability of iPSCs cultured on the scaffolds was assessed using an MTT assay on the 1st, 3rd, and 5th days in vitro (Figure 4B). SF50-10 and SF350-10 showed slightly decreased iPSC viability on the 1st day of culture compared to SF50-0 and SF350-0, respectively. However, this difference disappeared on the 3rd day, suggesting that all the scaffolds provide a suitable environment for IPSC proliferation. In fact, incorporating CNFs did not generate any statistically significant difference in the viability of iPSCs after the 1st day of culture (*p* > 0.05), independent of the pore size. However, iPSCs cultured on SF50-0 showed statistically higher viability than those cultured on both SF350-0 and SF350-10 on day 5 (*p* < 0.05 and *p* < 0.01, respectively). The scaffolds’ porous architecture was one of the major factors regulating cellular viability. SF50-0 had significantly higher interconnectivity than SF350-0 and SF350-10. Therefore, it provided a larger surface area for cellular proliferation. These findings were in line with the swelling ratio values of the scaffolds. SF50-0 had an almost 2.5 times higher swelling ratio than SF350-0 and SF350-10. This confirmed that the culture medium might penetrate better through SF50-0 and facilitate enhanced oxygen and nutrient transport throughout the scaffold.

Cardiomyogenic differentiation of iPSCs cultured on the scaffolds was assessed by RT-PCR through the expression of pluripotent-specific (Pouf5f1 and Nanog) and cardiac-specific (NKX2.5 and TNNT2) gene markers. Semiquantitative RT-PCR results are shown in Figure 4C. The scaffolds did not cause any statistically significant difference between the expression levels of Pou5f1 and Nanog markers on day 8 (*p* > 0.05). The trend was the same on day 14 for the iPSCs cultured on SF50-0, SF50-10, and SF350-10. However, iPSCs cultured on SF350-0 exhibited a substantial downregulation of the Pou5f1 and Nanog expression levels on day 14, indicating a loss of pluripotency. Independent of the scaffold type, cardiomyogenic induction of iPSCs resulted in the expression of the early cardiac marker NK2 homeobox 5 (NKX2.5) and the cardiac-specific structural gene marker cardiac troponin T (TNNT2) on day 8. However, iPSCs interacting with scaffolds did not differ in the expression levels of TNNT2 and NKX2.5 genes on day 8. However, on day 14, the expression levels of TNNT2 and NKX2.5 were the highest on SF350-0 compared to the other scaffolds. Cardiomyogenic differentiation of iPSCs was further assessed by immunostaining of cardiac-specific structural proteins, alpha sarcomeric actinin (α-SA), and cardiac troponin T (cTnT), at day 14. Images were depicted in Figure S8 and showed that iPSCs interacting with the SF350 scaffolds expressed higher amounts of α-SA and cTnT than SF50 scaffolds. In line with the RT-PCR results, SF350-0 stimulated the highest α-SA and cTnT expression from the iPSCs among all scaffolds.

To assess the cellular morphology of the iPSCs, SEM images were captured on the 1st, 8th, and 14th days of culture. See Figure 4D. These images provided further insight into cell-to-cell and cell-to-surface interactions. iPSCs appeared to interact with neighboring cells and formed compact dome-like colonies on the scaffolds on the 1st day. On day 8, for SF50-0 and SF350-0, the number of cells increased without showing an apparent difference in the cellular morphology. On the other hand, for SF350-10, well-spread, irregularly-shaped individual cells with multiple pseudopodia-like structures appeared on the 8th day of culture. In fact, they maintained their well-spread morphology on SF350-10 for up to 14 days. Colonies on SF50-0 and SF50-10 continued to grow until day 14 without significantly changing their cellular morphology. That said, incorporating CNFs seemed to induce cellular spreading both on SF50-10 and SF350-10. A possible explanation for these results could be the change in the wettability of scaffolds upon incorporating CNFs. Hydrophobic surfaces were reported to prohibit the spreading of iPSCs, lead to more cell-to-cell interactions (than cell-to-surface interactions), and cause clustering of iPSCs.^27−30^ Incorporating CNFs, however, increased the hydrophilicity of the SF scaffolds, as indicated previously.^12^ Thus, the enhanced cell spreading on SF50-10 and SF350-10 could be attributed to the increased hydrophilicity of the scaffolds. Interestingly, the cells on the surface of SF350-0 provided a flattened morphology on the 14th day of culture. This result supported the findings of other studies that cardiomyocytes differentiated from iPSCs (in vitro) exhibited flattened morphology.^31,32^ Indeed, our RT-PCR and immunostaining results also revealed that SF350-0 provided a more suitable environment for the cardiomyogenic differentiation of iPSCs than the other scaffolds.

In general, the pore architecture of scaffolds has been shown to affect iPSC differentiation. iPSCs tend to form clusters whose sizes depend on the pore size of the scaffold. An earlier study showed that larger cell clusters (around 450 μm) had a higher tendency to differentiate into cardiomyocytes, while smaller clusters (around 150 μm) differentiated into endothelial cells.^33^ The increased cardiomyogenic differentiation of iPSCs cultured on SF350-0 scaffolds might be attributed to the larger clusters formed on these scaffolds. On the other hand, incorporating CNFs into SF350-10 decreased its hydrophobicity, which might cause a decreased tendency of iPSCs to form large cell clusters and thus decrease the upregulation of cardiac-specific markers. Furthermore, the elastic modulus of scaffolds, enhanced by the incorporation of CNFs in this study, might affect iPSC differentiation.

Incorporating CNFs increased the modulus of scaffolds to around 40 kPa, whereas SF scaffolds without CNFs had an elastic modulus of about 30 kPa, independent of their pore sizes. Therefore, the increased modulus of SF350-10 due to incorporating CNFs was another possible reason (in addition to its decreased hydrophobicity) for the lack of upregulation in cardiac-specific markers compared to the SF350-0 scaffold.^34^ Taken together, SF350-0 provided a more suitable environment for the differentiation of iPSCs among all scaffolds investigated in this study. It is important to acknowledge that our findings offer valuable insights into the interactions between SF scaffolds and iPSCs. However, further research, including an evaluation of cardiomyocyte differentiation efficiency, is required to enhance our understanding of iPSC behavior on SF and SF/CNF scaffolds.

### 2.4 TENG Performance of the Scaffolds

SF/CNF scaffolds successfully mimicked the heart muscle tissue in terms of mechanical and electrical properties. That said, the SF350-0 better supported the cardiomyogenic differentiation of iPSCs compared to other scaffolds tested in this study. Therefore, SF350-0 was selected as one of the triboelectric layers in the TENG design. In addition, we demonstrated that the incorporation of CNFs into SF scaffolds altered their electronic behavior and thus provided an opportunity to utilize the SF350-10 scaffold across the SF350-0 as the counter-triboelectric layer.

**Figure 5:**
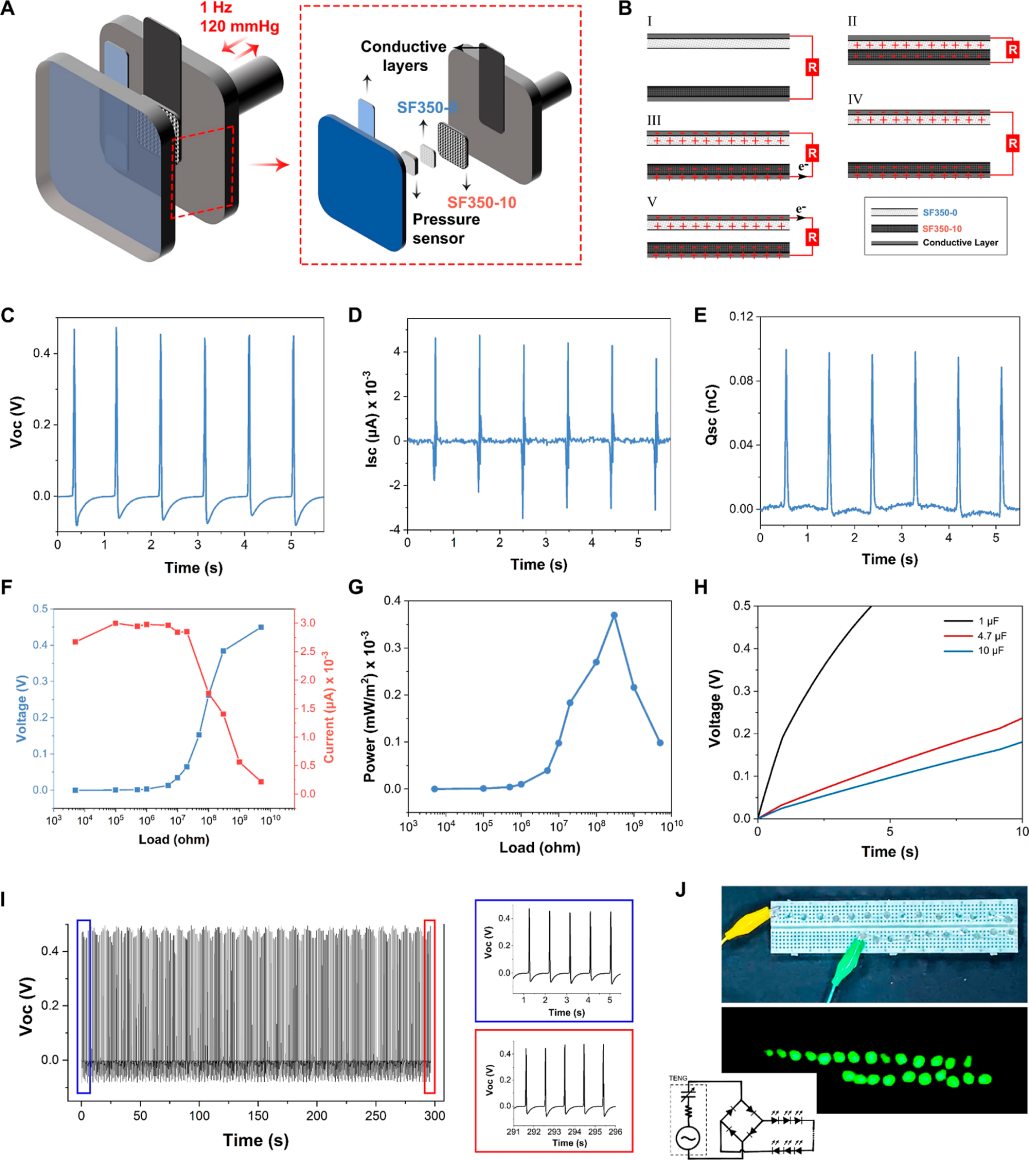
TENG performance of the scaffolds. (A) Schematic drawing of the TENG system to evaluate the performance of SF350 scaffolds under simulated cardiac motion. (B) Operating mechanism of the TENG used in this study. (C) *V*_OC_, (D) *I*_SC_, and (E) *Q*_SC_ obtained from the SF350-0 and SF350-10 electrodes. Output (F) voltage and current and (G) power obtained from SF350-0 and SF350-10 electrodes under different load resistors. (H) Charging of capacitors having different capacitance values using SF350-0 and SF350-10 electrodes. (I) Durability of SF350-0 and SF350-10 electrodes. (J) 24 serially connected LEDs illuminated using 4 × 4 cm SF350-0 and SF350-10 scaffolds as TENG electrodes.

A schematic drawing of the system used for the measurements of the scaffolds’ TENG performance is shown in Figure 5A (also see Video S1). SF350-0 and SF350-10 were mounted to the adjacent metallic grips using conductive adhesive layers. TENG performance data were acquired at a frequency of 1 Hz under 120 mmHg pressure (simulating the heart beating at rest). Figure 5B shows the operating mechanism of the TENG used in this study. Initially, there was no potential difference in the system (I). The surfaces of SF350-0 and SF350-0 were oppositely charged due to triboelectrification upon contact. Due to the insulative nature of SF, charges were preserved on the scaffold surfaces, and there was no practical electrical potential difference between the electrodes at this point (II). A potential difference started to build up when the two electrodes started to get separated. The electric potential increases with the increased distance between the electrodes, which then hits a maximum. Induced electrons started to flow from one electrode to the other due to the increased electrical potential (III). This electron flow results in the flow of a current that travels through the external circuit and reaches its maximum (IV). Closing the gap between the electrodes causes electron flow in the opposite direction (V). These periodic contacts and separations generate alternating currents.

The generated open circuit voltage (*V*_OC_), short circuit current (*I*_SC_), and short circuit (induced) charge (*Q*_SC_) of the TENG electrodes are shown in Figure 5C–E, respectively. Output voltages and currents were also measured with different load resistors connected to the circuit (Figure 5F). Output power with respect to resistance load was also calculated and is provided in Figure 5G. The scaffold/TENG system generated a maximum power output of 0.37 × 10^–3^ mW/m^2^ with a *V*_OC_, *I*_SC_, and *Q*_SC_ of 0.46 V, 4.5 nA, and 0.1 nC, respectively.

The TENG device, having SF350 scaffolds as electrodes, was used to charge capacitors to demonstrate its potential for energy storage, which might be beneficial to charge battery-operated mechanical devices such as left ventricular assist devices and pacemakers. The voltage changes of the capacitors, which have different capacitance values, are illustrated in Figure 5H. Performing the contact and separation cycles under simulated heart-beating conditions, the capacitors were charged with the rectified current.

The stability of the performance of the fabricated TENG device was also measured. Figure 5I shows the *V*_OC_ values during 300 s of tapping. The results showed that SF350 scaffolds were durable and generated stable *V*_OC_ values under the simulated heartbeat condition (120 mmHg tapping pressure at a frequency of 1 Hz). The TENG device, having 4 × 4 cm SF350 scaffolds as electrodes, was used to illuminate 24 serially connected LEDs, as shown in Figure 5J and Video S2.

The integration of TENG technology and cardiac tissue regeneration has several important implications. The use of TENGs in cardiac regeneration could provide a unique opportunity for real-time and in situ monitoring of tissue regeneration. By detecting electrical signals generated by the TENGs, it may be possible to assess the progression of cardiac tissue regeneration. This could lead to more effective and personalized treatment strategies for patients with heart failure. Another possible application of the scaffold/TENG system is the electrical stimulation of cells to enhance their cardiomyogenic differentiation. Though SF and SF/CNF scaffolds are expected to degrade in vivo in the long term, we expect and hypothesize that energy harvesting will still be active during the healing process and may not be required once tissue regeneration is complete. A similar approach could also be beneficial for other tissues under motion, such as hip, knee, and shoulder joints.

To summarize, we optimized the pore architecture of SF and SF/CNF scaffolds for the cardiomyogenic differentiation of iPSCs and, at the same time, generated electrical energy in response to mechanical stimulation. Combining TENGs with tissue engineering approaches would potentially pave the way for the development of a new generation of cardiac patches that can simultaneously provide mechanical support, electrical stimulation, and energy harvesting.

## 3 Conclusions

We successfully fabricated porous SF and SF/CNF scaffolds that mimic the mechanical and physical properties of cardiac tissue. Our findings showed that the total porosity, pore size, and interconnectivity of the scaffolds are all effective and crucial parameters in determining their mechanical strength, physical characteristics, degradation rate, and biological properties. In particular, the load-carrying mechanism, β-sheet content, degradation characteristics, and iPSC interactions of the scaffolds were all observed to be interrelated factors and regulated by their pore architecture and CNF content. Among the scaffolds fabricated in this study, the ones having a pore size, porosity, and pore interconnectivity of 379 ± 34 μm, 79 ± 1%, and 67 ± 1%, respectively, were optimal for cardiomyogenic differentiation of iPSCs. Incorporating CNFs brought an electrical conductivity of 0.021 ± 0.006 S/cm to SF scaffolds without changing their pore architecture. In addition, CNF incorporation allowed energy harvesting using these scaffolds as conjugate TENG electrodes. The TENG device having SF and SF/CNF scaffolds as counter electrodes generated a maximum power output of 0.37 × 10^–3^ mW/m^2^, with an open circuit voltage and short circuit current of 0.46 V and 4.5 nA, respectively, under a simulated cardiac motion. To conclude, we showed as a proof-of-concept that SF/CNF scaffolds could not only be used for the regeneration of cardiac tissue but also to generate power from the pulsatile nature of the cardiac motion for future applications.

## 4 Experimental Procedures

### 4.1 Materials

*Bombyx mori* (*B. mori*, silkworm) cocoons were purchased from Kozabirlik (Bursa, Turkey). CNFs (<100 ppm iron content), sodium carbonate (Na_2_CO_3_), lithium bromide (LiBr), 1,1,1,3,3,3-hexafluoro-2-propanol (HFIP), protease type XIV (>3.5 units/mg), and methanol (CH_3_OH) were purchased from Sigma-Aldrich. NaCl was purchased from Isolab. Dulbecco’s modified Eagle’s medium, penicillin–streptomycin, fetal bovine serum (FBS), and trypsin–EDTA were purchased from Biosera. 3-(4,5-Dimethyl-2-thiazolyl)-2,5-diphenyl-2*H*-tetrazolium bromide (MTT), dimethyl sulfoxide (DMSO), glutaraldehyde, and hexamethyldisilazane (HMDS) were purchased from Glentham, Genaxxon Bioscience, Merck, and Sigma, respectively.

### 4.2 Fabrication of Porous SF/CNF Scaffolds

SF from *B. mori* cocoons was extracted by dissolving the outer sericin layer in a 0.02 M Na_2_CO_3_ solution for 30 min at 80 °C. The extracted SF was dried for 12 h, dissolved in a 12 M LiBr solution for 4 h at 60 °C, and dialyzed against distilled water for 4 d. The dialyzed SF solution was frozen at −20 °C for 24 h and lyophilized using Christ Alpha 2–4 LDplus. HFIP and NaCl particles were used as the solvent and the porogen agent, respectively. NaCl particles were sieved to obtain 50–90 and 350–425 μm particle size ranges, as per the ASTM-E1638 standard, and weighted to obtain a NaCl/SF weight ratio of 10:1. Prior to scaffold fabrication, CNFs were treated in a 1:1 HNO_3_/H_2_SO_4_ (v/v) solution for 24 h and washed several times with a copious amount of deionized (DI) water. In the meantime, SF was dissolved directly in HFIP to obtain a 4% (wt/v) SF/HFIP solution. For the samples containing CNFs, first, 10 wt % CNF was dispersed in HFIP, followed by dissolving the SF in this mixture to obtain the SF/HFIP/CNF mixture. Afterward, NaCl particles were incorporated into the HFIP/CNF/SF mixture, and the mixture was placed inside a fume hood for 24 h to evaporate the HFIP. Once the HFIP was removed, samples were placed in methanol for 24 h to induce crystallization. Finally, the scaffolds were washed with DI water for 4 d to dissolve NaCl particles.

### 4.3 Characterization of the Scaffolds

A scanning electron microscope (FEI Nova Nano SEM 430) was used to image cross-sections of the scaffolds and cellular morphologies. A Quorum SC7640 high-resolution sputter coater was used to coat a thin gold layer onto the scaffolds to create an electrically conductive path prior to SEM imaging.

Scaffolds were scanned with μ-CT (Bruker μ-CT, 1275), where the pixel size was 7 μm per pixel and a 25 kV voltage was used without any filter. Samples were rotated throughout 360° via 0.2° per step. Porosity and interconnectivity data were calculated using CTAn software (Bruker μ-CT). Also, CTVox software was used to perform the 3D visualization of the scaffolds. The interconnectivity of the samples was calculated according to the protocol established by Fostad et al.^35^ The volume of interest (VOI) was subjected to the 3D region of interest shrink-wrap operation using CTAn software. The percentage of interconnectivity was calculated using eq 1, where *V* is VOI, *V*_sw_ is VOI after shrink-wrap processing, and *V*_m_ is the volume of the scaffold.
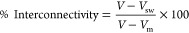
1

For the swelling ratio measurements, the scaffolds were cut into rectangular-shaped samples with an approximately equivalent mass (60 ± 5 mg) and placed inside phosphate-buffered saline (PBS). Swollen scaffolds were weighed at predetermined time points after wiping excess liquid at their surfaces. Swelling ratios were calculated using eq 2, where *W*_0_ and *W*_S_ are the masses of the dry and swollen scaffolds, respectively.
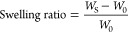
2

Scaffolds were scanned in the 4000–400 cm^–1^ range with 4 cm^–1^ resolution using a PerkinElmer 400 Fourier transform infrared spectrometer in the attenuated total reflection configuration. Background spectra were subtracted from the obtained reflectance data. To identify the secondary structure of the scaffolds, deconvolution was applied to the amide I region (1595–1705 cm^–1^) using OPUS5.0 software. Firstly, second derivatives were calculated for the original spectra in the amide I region with a nine-point Savitsky-Golay smoothing filter to determine the number and positioning of the bands.^36^ Then, the baseline was subtracted from the original band, and the Gaussian function was used for the curve fitting. Areas under single bands were used to determine the fraction of the secondary structure elements.^36^ Three measurements were carried out for each sample group, and average values were reported.

Uniaxial tensile tests (Shimadzu AGS-X) were performed on 1 × 5 cm-sized samples as per the ASTM 638 standard. A gauge length of 2 cm and a displacement rate of 1 mm s^–1^ was used for the experiments. The load cell of the test instrument had a 1 kN capacity. The mechanical properties of the scaffolds were tested in the wet condition at 37 °C.

Impedance measurements were conducted using symmetric stainless-steel electrodes within Swagelok cells. The HP 4194a Impedance/Gain-Phase Analyzer was used to conduct electrochemical measurements between 100 Hz and 40 MHz. Measurements were performed on four different samples for each scaffold. The HP 4194a Impedance/Gain-Phase Analyzer was used to measure the capacitances. The dielectric constant of the scaffolds was calculated using eq 3, where *C* indicates the measured capacitance, ε_0_ is the permittivity of vacuum, *A* is the area, *d* is the thickness, and ε_r_ shows the dielectric constant of the samples.
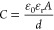
3

The porosity of the scaffolds was calculated based on the capacitance measurements of the samples. The measured capacitance values of the scaffolds were included in the capacitive contributions of the air within the pores and the surrounding SF matrix. The dielectric constant of the SF matrix was determined by measuring the dielectric constant of a SF film (4 wt/v %) without any porosity. The dielectric constant of air was taken as 1. The ratios of air and SF in the scaffolds were determined according to the capacitive contributions calculated using the dielectric values of the components. The calculated air ratio was presented as the scaffold’s porosity. Following refs (37) and (38), the electrical conductivity of the SF/CNF films and scaffolds was measured.

Semi-quantitatively simulated pore structures were created in Blender software, where the pore structures were drawn considering the measured porosity, pore size, and interconnectivity values of the scaffolds. These drawings were used to explain how a scaffold’s pore architecture affects its strength.

### 4.4 Biodegradation of the Scaffolds

Scaffolds having an approximately equivalent mass (60 ± 5 mg) were incubated in a 12 mL solution of 1 U/mL protease XIV prepared in sterile PBS (pH: 7.4). The enzyme solutions were replenished every 3 days. Scaffolds were rinsed in DI water at designated time points and prepared for the characterizations. The thickness of the scaffolds was measured from arbitrarily selected regions in their cross-sectional SEM images to repost reduction-in-thickness measurements. Three different measurements from three different samples were performed for each scaffold. The absorbance values of the enzyme solutions were measured at 280 nm (the as-prepared enzyme solution was used as a control). Weight loss calculations were carried out using eq 4, where *W*_1_ and *W*_2_ are the dry masses of the scaffolds prior to and after degradation, respectively.
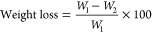
4

For mechanical testing, 5 × 1 cm rectangular-shaped samples were degraded in 1 U/mL enzyme solution for 6 days. Afterward, samples were rinsed in DI water, and the uniaxial tensile tests were performed, as detailed in the Experimental Procedure section.

### 4.5 Biological Characterizations

#### 4.5.1 Sterilization of the Scaffolds

Scaffolds were sterilized with 70% (v/v) ethanol three times, followed by UV radiation each for 15 min. Afterward, they were incubated in Iscove’s Modified Dulbecco’s Medium (IMDM, Biosera) supplemented with 5% (v/v) penicillin/streptomycin (P/S, Biosera) for 3 days. Samples were equilibrated with growth medium consisting of IMDM supplemented with 20% FBS (Biosera), 1% non-essential amino acid (NEAA) (Minimum Essential Medium, 100X, Biosera), 1% P/S (100X, Biosera), 0.1 mM β-mercaptoethanol (β-ME) (Sigma), and 1000 U/mL leukemia inhibitory factor (LIF) (ORF Genetics) for 24 h prior to cell culture experiments.

#### 4.5.2 Murine-Induced Pluripotent Stem Cell Culture and Cardiomyocyte Differentiation

Murine-induced pluripotent stem cells (iPSCs) (clone TαP4) were cultured on six-well plates coated with 0.2% gelatin in a growth medium at 37 °C and 5% CO_2_. The growth medium was replenished every other day. When the confluency reached 60–70%, cells were trypsinized and seeded onto the sterile scaffolds. IPSCs seeded on the scaffolds were cultured with the growth medium. When cells reached confluency, the medium was changed to a differentiation medium consisting of IMDM supplemented with 20% FBS, 1% NEAA, 1% P/S, 0.1 mM β-ME, and 50 μg/mL l-ascorbic acid-2-phosphate (AA) (Sigma) to initiate cardiac differentiation.^30,38^ The differentiation medium was replenished every day. Once beating cells were observed, the FBS content of the differentiation medium was lowered to 5%, and AA was removed from the differentiation medium. Cells were cultured with this medium until the 14th day of culture. The medium was changed every day.^39^

#### 4.5.3 Cell Viability

The viability of iPSCs on the scaffolds was investigated using a 3-(4,5-dimethyl-2-thiazolyl)-2,5 diphenyl-2*H*-tetrazolium bromide (MTT) (Sigma) assay at the 1st, 3rd, and 5th days of culture. The growth medium was replaced by 150 μL of 0.5 mg/mL MTT reagent prepared in the growth medium, and cells were incubated for 4 h at 37 °C. After removing the MTT reagent, dimethyl sulfoxide (DMSO) was added, and samples were gently agitated on the shaker for 5 min at room temperature to dissolve formazan crystals. After transferring 100 μL of the dissolved formazan into 96-well plates, the optical densities of the solutions were measured using a microplate absorbance reader (iMark Microplate Absorbance Reader, Bio-Rad) at 570 nm (reference wavelength—750 nm).

#### 4.5.4 Cell Morphology Analysis

The morphology of iPSCs grown on the scaffolds was analyzed on the 3rd day of culture. Initially, cells were fixed with 3% (v/v) glutaraldehyde (Sigma), followed by sequential dehydration using 30, 50, 70, 90, and 100% absolute ethanol for 10 min each and finally treated with hexamethyldisilazane (HMDS, Sigma) overnight.^40^ Prior to SEM imaging, scaffolds were sputter-coated with a thin platinum layer.

#### 4.5.5 Immunocytochemistry Staining

At 14 days of culture, samples were rinsed with PBS, and cells were fixed with 4 wt % paraformaldehyde (PFA) at room temperature for 30 min. After fixation, cells were permeabilized with 0.1% Triton X-100 for 5 min and blocked with 1 wt % bovine serum albumin containing 22.52 mg/mL glycine prepared in PBS for 30 min. Then, samples were incubated in anti-sarcomeric α-actinin (1:50 dilution, ab 9465, Abcam) and monoclonal anti-cardiac troponin T (cTnT) (1:25 dilution, ab33589, Abcam) primary antibodies for 4 h at room temperature.^41^ Afterward, samples were rinsed with PBS and incubated in Alexa Fluor 555 (1:1000, ab150118, Abcam) and Alexa Fluor 488 (1:1000, ab150113, Abcam) secondary antibodies for 1 h to stain α-actinin and cTnT, respectively. The actin cytoskeletons of the cells were stained with Alexa Fluor 488 Phalloidin (Thermo Fisher). The nuclei of cells were counterstained with 25 mM Hoechst (Abcam, ab228551). Images were acquired using a confocal microscope (DMI 8, Leica), and processed using Leica LASX software.

#### 4.5.6 Polymerase Chain Reaction

IPSCs were seeded on sterile scaffolds at a density of 5 × 10^4^ cells/cm^2^. The expression levels of Nanog and Pou5f1 as specific pluripotent genes and Nkx-2.5 and TnnT2 as specific cardiac genes were quantified relative to the level of glyceraldehyde-3-phosphate dehydrogenase (GAPDH) on the 8th and 14th day of culture using the real-time polymerase chain reaction (RT-PCR) analysis. Total RNA was initially extracted using a High Pure RNA isolation kit (Roche, Switzerland) according to the manufacturer’s instructions. Afterward, cDNA was synthesized through reverse transcription reaction using Transcriptor First Strand cDNA kit in a thermal cycler (Bio-rad) at 25 °C for 10 min, 50 °C for 60 min, and 85 °C for 5 min. RT-PCR analysis was conducted with primer-probe sets for each target gene using the Lightcycler 480 master kit (Roche) according to the protocol provided by the manufacturer under cycling conditions of pre-incubation at 95 °C for 10 min, 45 amplification cycles of denaturation at 95 °C for 10 s, and annealing at 53 °C for 15 s, followed by elongation at 72 °C for 1 s, and cooling at 40 °C for 30 s. Three samples were examined for each group, and the measurements were repeated in triplicate. The relative expression levels of each gene were determined according to the 2^–ΔΔ*Cp*^ method.^42^

### 4.6 TENG Measurements

A linear actuator operating at a frequency of 1 Hz was used for TENG measurements. The tapping pressure (12 mmHg) and frequency were adjusted to represent a resting human heartbeat. The open-circuit voltage (*V*_OC_), short-circuit current (*I*_SC_), and short-circuit charge (*Q*_SC_) of the fabricated TENGs were measured using a Keithley 6514/E-Electrometer. A LabView code was used to collect the data.

### 4.7 Statistical Analysis

Cell viability and gene expression data were presented as mean ± standard deviation and statistically analyzed by ANOVA and Tukey post hoc test. The statistically significant difference between the experimental groups was determined using p values (ns, *p* ≥ 0.05; *p < 0.05; **p ≤ 0.01; ***p ≤ 0.001).

## Supporting Information Available

System used for the measurements of the scaffolds’ TENG performance (MOV)
TENG device, having 4 × 4 cm SF350 scaffolds as electrodes, used to illuminate 24 serially connected LED (MOV)
Photographs, secondary structure component percentages, semi-quantitatively simulated pore structures, frequency-dependent impedance behavior, electrical conductivity, after-degradation photographs, SEM images and secondary structure components of scaffolds, and immunostaining proteins of iPSCs (PDF)

## Supplementary Material

am3c08601_si_001.mov

am3c08601_si_002.mov
