# Supplementary material for: Multifunctional Silk Fibroin/Carbon Nanofiber Scaffolds for In Vitro Cardiomyogenic Differentiation of Induced Pluripotent Stem Cells and Energy Harvesting from Simulated Cardiac Motion
Source: ACS Appl Mater Interfaces. 2023 Aug 29;15(36):42271–83. doi: 10.1021/acsami.3c08601 (PMC10510024; doi:10.1021/acsami.3c08601)
Supplement: Supplementary file 3 — am3c08601_si_003.pdf [file am3c08601_si_003.pdf]

Supporting Information

Multifunctional Silk Fibroin/Carbon Nanofiber Scaffolds for *in vitro* Cardiomyogenic Differentiation of iPSCs and Energy Harvesting from Simulated Cardiac Motion

Yiğithan Tufan[1], Hayriye Öztatlı[2], Doga Doganay[1], Arda Buyuksungur[3], Melih Ogeday Cicek[1], İpek Tuğçe Döş[1], Çağla Berberoğlu[1], Husnu Emrah Unalan[1], Bora Garipcan[2], Batur Ercan[1, 4, 5]*

[1] Department of Metallurgical and Materials Engineering, Middle East Technical University, 06800, Çankaya, Ankara, Turkey

[2] Institute of Biomedical Engineering, Boğaziçi University, 34684, İstanbul, Turkey

[3] Department of Basic Medical Sciences, Faculty of Dentistry, Ankara University, Ankara, Turkey

[4] Biomedical Engineering Program, Middle East Technical University, 06800, Çankaya, Ankara, Turkey

[5] BIOMATEN, Center of Excellence in Biomaterials and Tissue Engineering, Middle East Technical University, 06800, Çankaya, Ankara, Turkey

*Email: baercan@metu.edu.tr

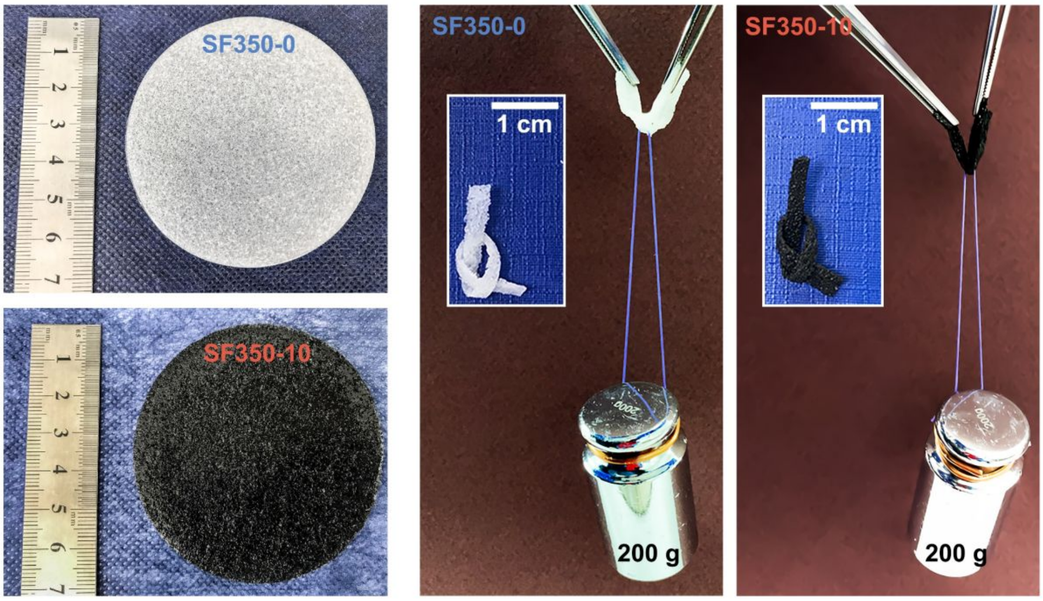

Figure S1. Photographs showing that SF350-0 and SF350-10 scaffolds are flexible and suturable.

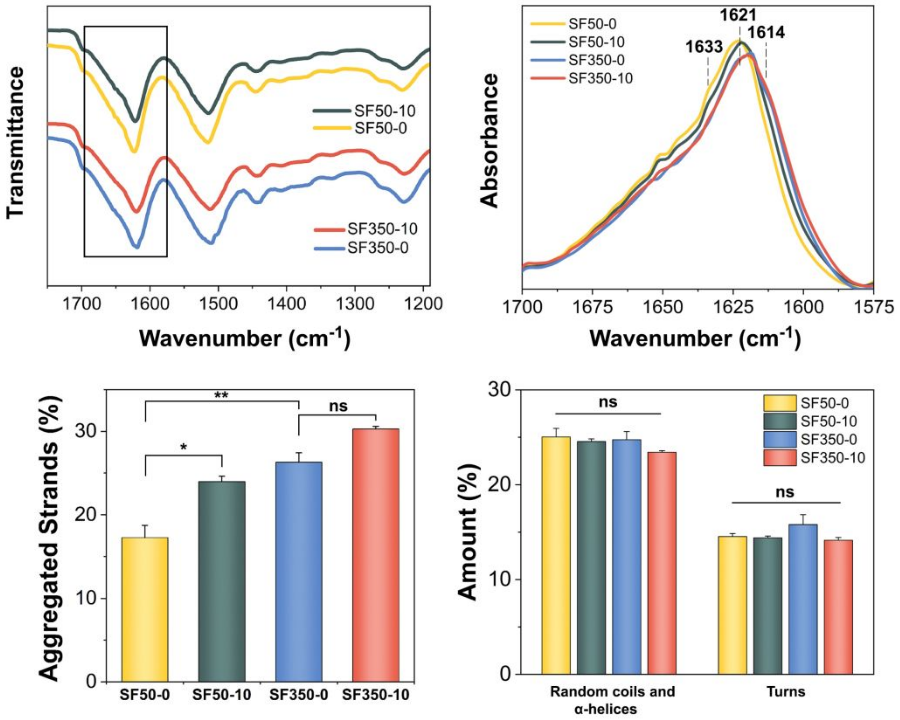

Figure S2. Deconvolution of the amide I regions in the FTIR spectra of the scaffolds comparing their aggregated strand, random coil/α-helices, and turn contents. ns: not significant, $*p < 0.05$, $**p < 0.01$.

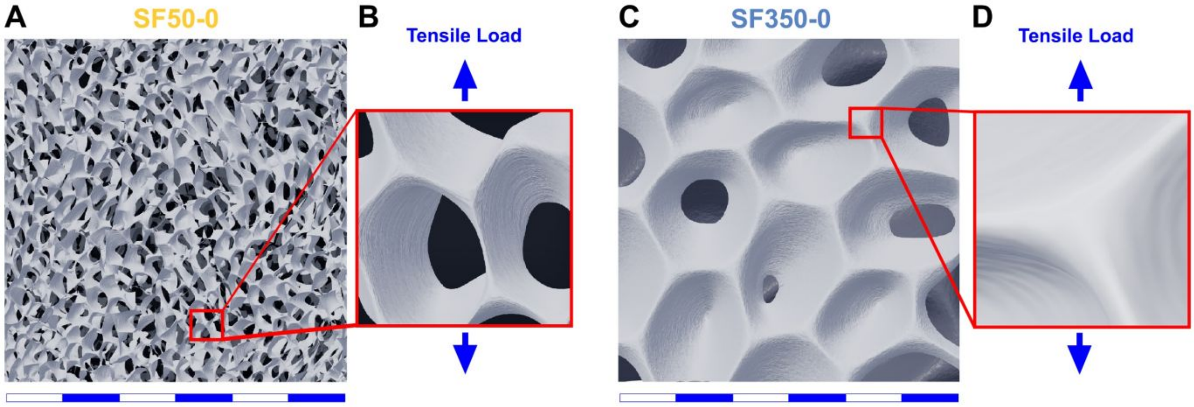

Figure S3. Semi-quantitatively simulated pore structures of A) and B) SF50-0, and C) and D) SF350-0.

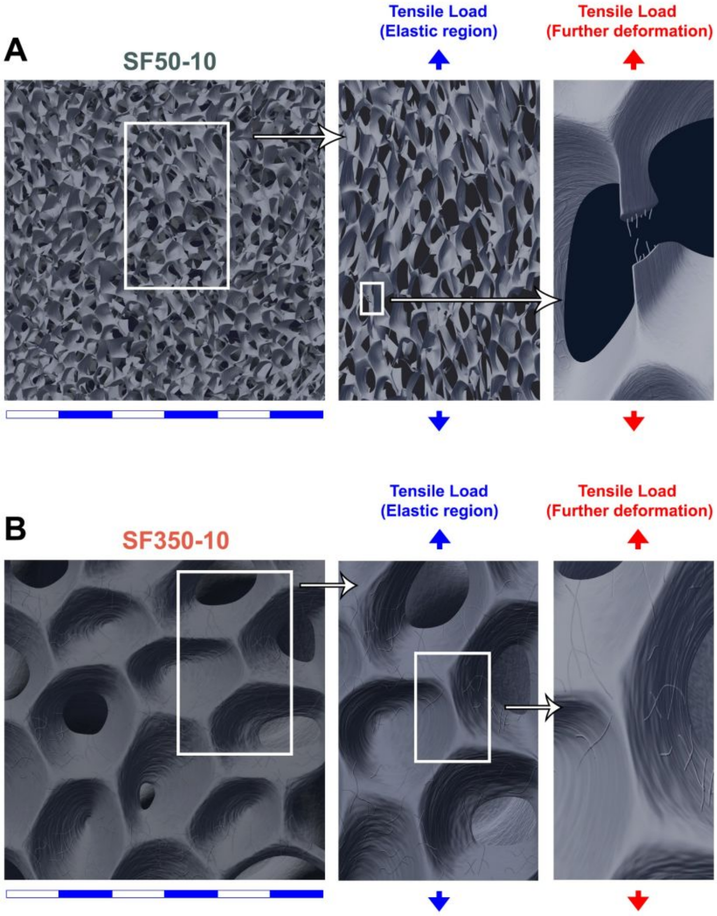

Figure S4. Semi-quantitatively simulated pore structures of A) SF50-10 and B) SF350-10.

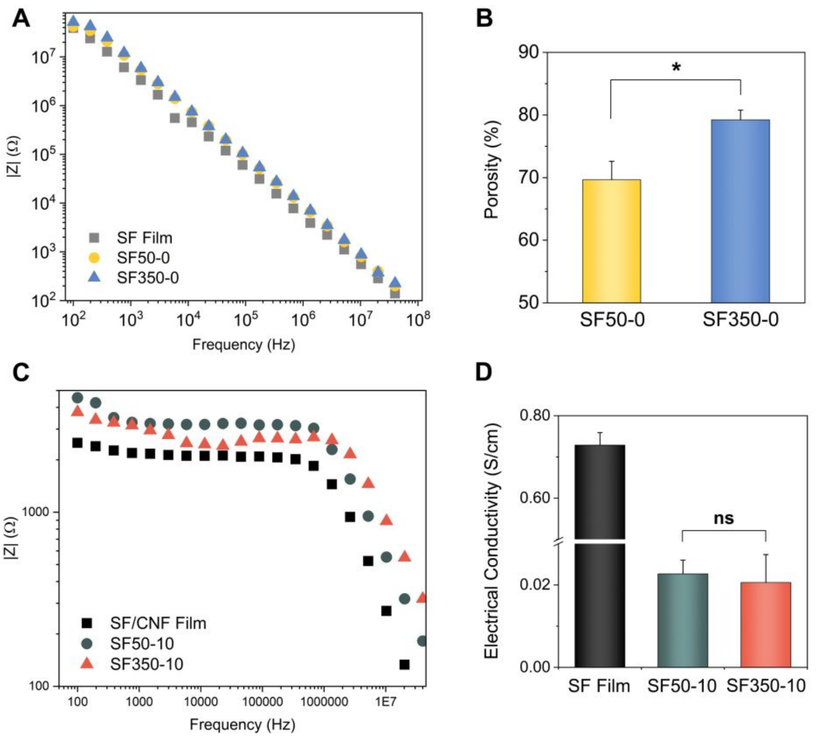

Figure S5. A) Frequency-dependent impedance behaviour of the SF film, SF50-0, and SF350-0 scaffolds. B) Porosity percentages of SF50-0 and SF350-0 calculated according to their dielectric constants. C) Frequency-dependent impedance behaviour and D) electrical conductivity values of the SF/CNF film, SF50-10, and SF350-10 scaffolds. ns: not significant, $^*p < 0.05$.

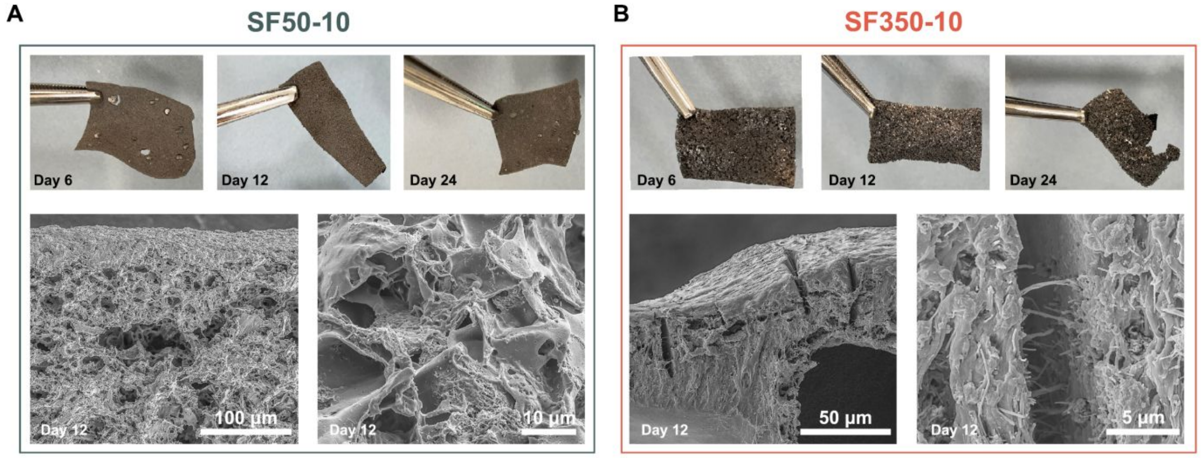

Figure S6. Photos and SEM images of A) SF50-10 and B) SF350-10 captured at various incubation times.

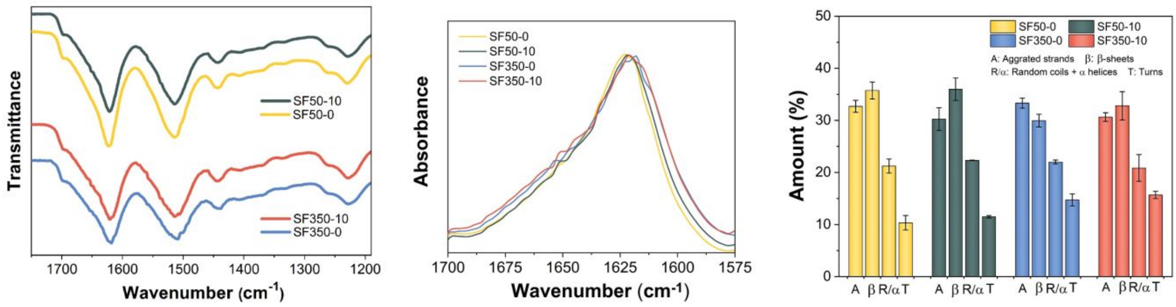

Figure S7. Deconvolution of the amide I regions in the FTIR spectra of the scaffolds at the 6th day of incubation and the comparison of the percentages of their secondary structural components.

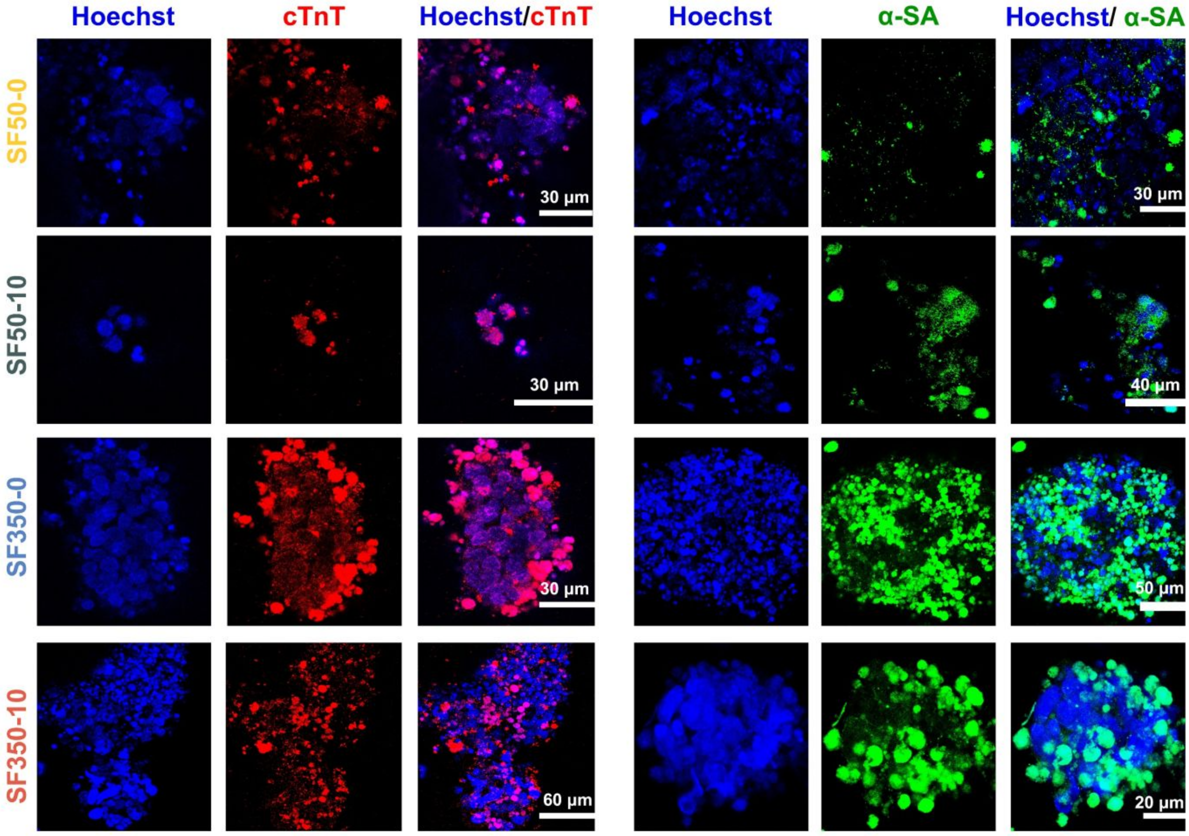

Figure S8. Immunostaining of alpha sarcomeric actinin (α-SA) and cardiac troponin T (cTnT) proteins expressed from iPSCs cultured on different scaffolds. Images were obtained at the 14th day of culture.

Supp. Video 1 - Data acquiring.MOV

Supp. Video 1

Supp. Video 2 - Led Video.MOV

Supp. Video 2
